# RACIAL AND NATIVITY DIFFERENCES IN ADVERSITY PROFILES AMONG MIDDLE-AGED AND OLDER ADULTS

**DOI:** 10.1093/geroni/igad104.0997

**Published:** 2023-12-21

**Authors:** Man Guo, Yi Wang, Kara Carter

**Affiliations:** University of Iowa, Iowa City, Iowa, United States; University of Iowa, Iowa City, Iowa, United States; University of Iowa, Iowa City, Iowa, United States

## Abstract

Cumulative adversity theory explains how distribution of resources and stress accumulate over time, resulting in divergent later life outcomes. Focusing on the nexus of race and nativity, this study examined profiles of adversity (i.e., the overall typological patterns of adversity) and their mental health implications in four groups of middle-aged and older adults: native-born whites, native-born ethnic minorities, foreign-born whites, and foreign-born ethnic minorities. Data were from the 2018 psychosocial assessment of the HRS (N = 5,534). Latent class analysis (LCA) with multigroup analyses were employed to identify typological patterns of ten adversity indicators and to compare the latent structures and class prevalence across the race/nativity groups. Regressions were used to examine the associations between adversity profiles and three outcomes: depression, pessimism, and life satisfaction. adversity profiles emerged: low adversity (60.05%), low human capital (15.69%), socially marginalized (13.67%), and neighborhood adversity (10.59%). These adversity profiles were structurally the same for the four race/nativity groups, but the class prevalence varied across the groups. Overall, having low adversity profile was associated with the best mental health outcomes. However, the mental health implications of each adversity profile varied by outcomes and by race and/or nativity. Findings highlight the importance of examining heterogeneity in adversity and its mental health implications among disadvantaged populations and the need of developing tailored programs to address unique needs of different minority populations.

